# Effect of ischemic compression on myofascial pain syndrome: a systematic review and meta-analysis

**DOI:** 10.1186/s12998-022-00441-5

**Published:** 2022-09-01

**Authors:** Wei Lu, Jiong Li, Ye Tian, Xingang Lu

**Affiliations:** 1 Department of Nursing, Longhua Hospital Affiliated to Shanghai University of Traditional Chinese Medicine, Shanghai, 200032 People’s Republic of China; 2 Department of Hepatobiliary Surgery, Longhua Hospital Affiliated to Shanghai University of Traditional Chinese Medicine, Shanghai, 200032 People’s Republic of China; 3 Department of Rehabilitation, Baoshan Branch, Ren Ji Hospital, School of Medicine, Shanghai Jiao Tong University, Shanghai, 200436 People’s Republic of China; 4 Department of Traditional Chinese Medicine, Huadong Hospital Affiliated to Fudan University, Shanghai, 200040 People’s Republic of China

**Keywords:** Ischemic compression, Massage, Manual therapy, Myofascial pain, Trigger point

## Abstract

**Background:**

Myofascial pain syndrome (MPS) is a condition with local and referred pain characterized by trigger points (taut bands within the muscle). Ischemic compression is a noninvasive manual therapy technique that has been employed for the treatment of MPS in past decades. However, little attention has been devoted to this topic.

**Objectives:**

The present review was designed to explore the efficacy of ischemic compression for myofascial pain syndrome by performing a descriptive systematic review and a meta-analysis to estimate the effect of ischemic compression on MPS.

**Methods:**

A systematic review and meta-analysis concerning randomized controlled trials (RCTs) with myofascial pain subjects who received ischemic compression versus placebo, sham, or usual interventions. Five databases (PubMed, The Cochrane Library, Embase, Web of Science, Ovid) were searched from the earliest data available to 2022.1.2. The standardized mean difference (SMD) and the 95% confidence interval (CI) were used for statistics. Version 2 of the Cochrane risk of tool 2 (RoB 2) was used to assess the quality of the included RCTs.

**Results:**

Seventeen studies were included in the systematic review, and 15 studies were included in the meta-analysis. For the pressure pain threshold (PPT) index, 11 studies and 427 subjects demonstrated statistically significant differences compared with the control at posttreatment (SMD = 0.67, 95% CI [0.35, 0.98], P < 0.0001, I^2^ = 59%). For visual analog scale (VAS) or numeric rating scale (NRS) indices, 7 studies and 251 subjects demonstrated that there was no significant difference between ischemic compression and controls posttreatment (SMD = − 0.22, 95% CI [− 0.53, 0.09], *P* = 0.16, I^2^ = 33%).

**Conclusion:**

Ischemic compression, as a conservative and noninvasive therapy, only enhanced tolerance to pain in MPS subjects compared with inactive control. Furthermore, there was no evidence of benefit for self-reported pain. The number of currently included subjects was relatively small, so the conclusion may be changed by future studies. Big scale RCTs with more subjects will be critical in future.

## Introduction

Myofascial pain syndrome (MPS) is a type of musculoskeletal pain that commonly occurs in muscle and surrounding fascia [1, 2]. MPS was first descripted by Drs Janet Travel and David Simons [3]. MPS has a high prevalence of 85% among patients complaining chronic pain in a survey [4] and 9% of total patients in another survey [5]. One or more trigger points found in the related muscle and fascia are the main characteristic of MPS [6]. The trigger point refers to a specific sensitive zone or point, tender region or a taut band in the skeletal muscle [2]. When this area or this point is under pressure, stretching or contraction, the pain can be further aggravated. Additionally, MPS can result in other pain-related symptoms, such as limited range of motion, skin blood flow response [7] and weakness [8]. Chronic or acute muscle injury, repetitive muscle overuse contributes to the cause of MPS [9]. The excess production of proinflammatory cytokines and other circulating biomarkers, even vascular biomarkers elicits pain in MPS subjects [10, 11].

Treatment of MPS includes dry needling, medication injection, stretching exercise, low laser therapy, and manual therapy [1]. Manual therapy includes a wide variety of techniques, such as chiropractic, massage, mobilization, muscle energy, and counter stain techniques [12]. Among them, ischemic compression, also known as manual pressure release [13] or trigger point release massage [14, 15], is a type of manual therapy that is commonly applied for MPS treatment [16, 17]. Ischemic compression is characterized by continuous compression or sustained pressure at several times to the trigger point or approximate regions commonly with a duration of 30–90 s (Specifically 30, 60 or 90 s) [18]. This pressure can elicit a local ischemia and further blood reperfusion, which results in the increase of muscle metabolism [19]. A systematic review published in 2015 and including relevant randomized controlled trials (RCTs) until 2013 demonstrated that there was moderate evidence that ischemic compression had a beneficial effect on MPS [20]. However, this review only included qualitative synthesis, and no quantitative synthesis was performed due to a lack of data at that time. Another review also pointed out that manual therapy had an effect on myofascial pain related to temporomandibular disorders compared with sham treatment, but this review did not include ischemic compression RCTs [21]. Most recently, during our work, a meta-analysis demonstrated that ischemic compression promoted the recovery of range of motion in MPS subjects [22]. However, although pain is the primary syndrome of MPS subjects, no analysis or conclusion was made regarding the effect of ischemic compression on the pain of MPS subjects in the meta-analysis [22]. In present systematic review and meta-analysis, we investigated the effect of ischemia compression on myofascial pain syndrome focusing on the pain experience of subjects.

## Materials and methods

This systematic review was structured following the statement of PRISMA (Preferred Reporting Items for Systematic Reviews and Meta-Analyses) [23] and Cochrane review guidance [24] and was registered at Inplasy (INPLASY202240066).

### Data sources and search strategy

The PubMed, The Cochrane Library, Excerpta Medica (Embase), Web of Science, Ovid Medical Literature Analysis and Retrieval System Online (OVID) databases were searched from the earliest data up to 2022/1/2. The search strategy included the following terms: (Massage OR Chiropractic OR manual therapy OR tuina OR Shiatsu OR Acupressure OR Ischemic compression OR myofascial release) AND (Myofascial pain OR Trigger point) AND (Randomized Controlled Trials OR trial OR placebo OR groups OR control OR Random*). Furthermore, some “grey” literature was retrieved by manual checking the reference lists in relevant reviews, trials or conference literature. Trials ongoing were also manually checked from the website www.clinicaltrial.gov. The language was set as English.

### Selection and exclusion criteria

The present systematic review included articles that met the following PICOS criteria: (1) patients: confirmed diagnosis of MPS according to the established criteria by Simon et al. [25, 26]; (2) intervention: Ischemic compression therapy should be administered alone or as the primary intervention combined with the usual intervention; (3) comparison or control: inactive comparison of sham or placebo, or active comparison using other usual intervention; (4) outcomes: pain is the primary outcome, and other indices that reflect the quality of life or other MPS-related symptoms are secondary outcomes; and (5) study: only RCTs.

The exclusion criteria were as follows: (1) other chronic pain conditions without trigger points or myofascial pain; (2) sufficient data cannot be obtained from RCT for example data is shown in figures and authors could not be reached; (3) comparison was set as another type of massage or manual therapy; or (4) ischemic compression is part of physical therapy, or the absence of proper control, which makes ischemic compression the only difference.

### Screening and data extraction

Two authors (WL and JL) independently screened all literatures. Duplicate titles and abstracts were removed initially, and the most recent was retained. If the title or abstract met the inclusion criteria, the full text of the article was downloaded and carefully reviewed. Discrepancies were resolved by a senior investigator (XGL). The following data were extracted from the selected studies and organized into spreadsheets: general information, subjects, ischemic compression procedures and controls, durations, effect sizes, outcomes, follow-up periods, and adverse events.

### Risk of bias assessment

Two independent reviewers (WL and JL) assessed the quality and bias of the included meta-analyses using the randomized trial bias risk tool 2 (RoB 2) revised by the Cochrane collaboration [27]. If a discrepancy existed, then the question was subjected to a third reviewer (TY). Using RoB 2 tools, each standard has five results: "yes", "probably yes", "probably no", "no" and "no information". The overall bias was automatically generated by the RoB 2 tool, and the authors made their own judgments based on the results.

### Data analysis

A meta-analysis was used to combine evidence from included RCTs when available of pain indices such as visual analog scale (VAS) or numeric rating scale (NRS), pressure pain threshold (PPT). Revman Manager 5.3 software (Cochrane Corporation, Texas, USA) was employed for data analysis. The standard mean difference (SMD) and respective 95% confidence interval (CI)s were calculated for the effect measure of continuous outcomes. I^2^ greater than 50% was considered significant for heterogeneity. A *P* value < 0.05 was considered statistically significant. A fixed or random effects model was chosen based on clinical heterogeneity based on the Cochrane Handbook [28]. Sensitivity analyses and subgroup analyses were planned following below items: inactive/active control, duration of treatment, location of compression, male/female of subjects.

## Results

### Study flow of literature search

**Fig. 1:**
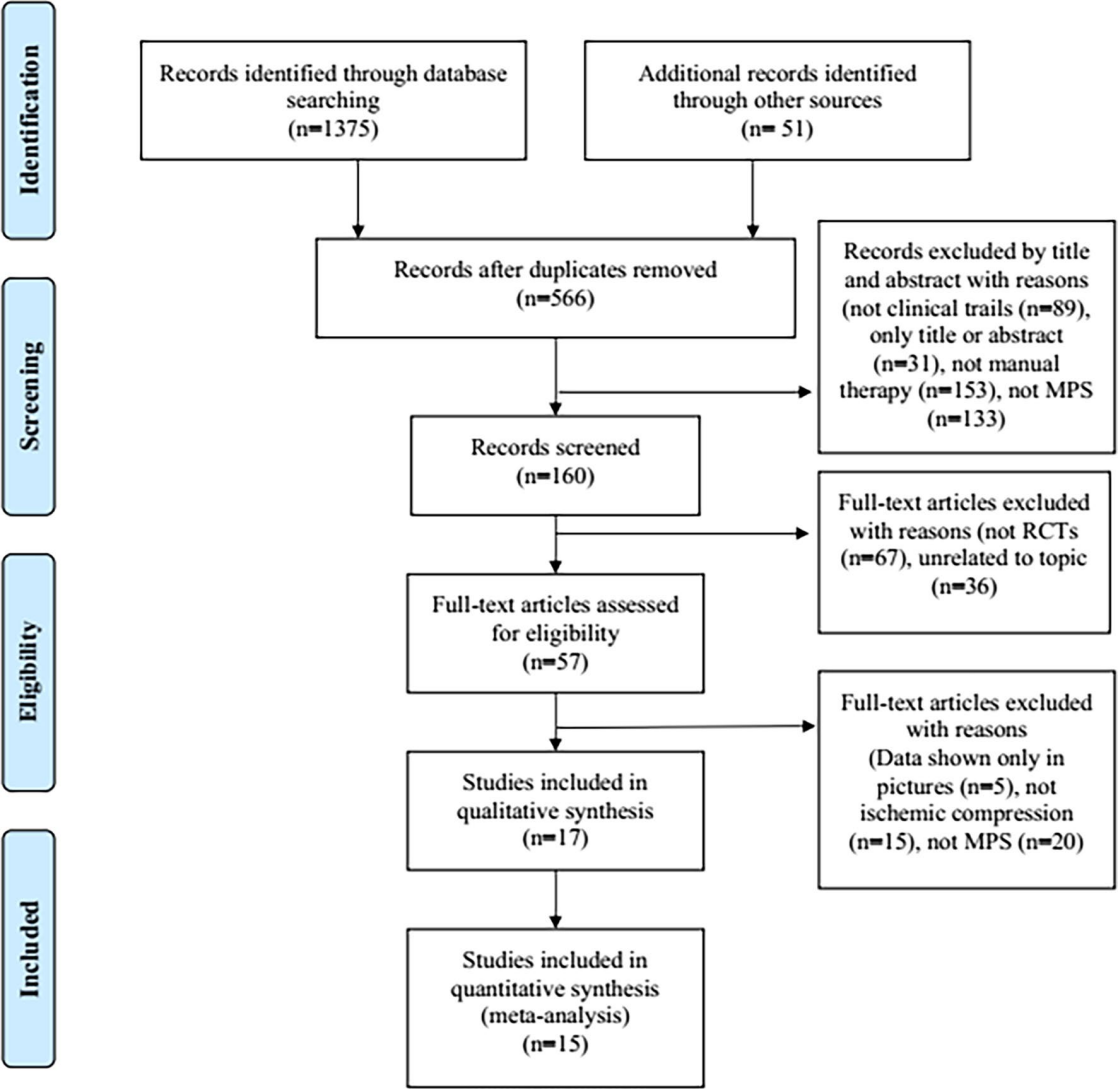
Flow diagram of this systematic review and meta-analysis study

In the preliminary search, 1426 studies were identified. After excluding duplicated studies, 566 studies remained. Next, 406 studies were removed by title and abstract reading. After full text review, 103 studies were excluded for reasons (not RCTs and unrelated topic). Among the remaining 57 studies, 40 studies were further excluded. The flow diagram is shown in Fig. 1.

### Description of RCTs and quality

**Fig. 2:**
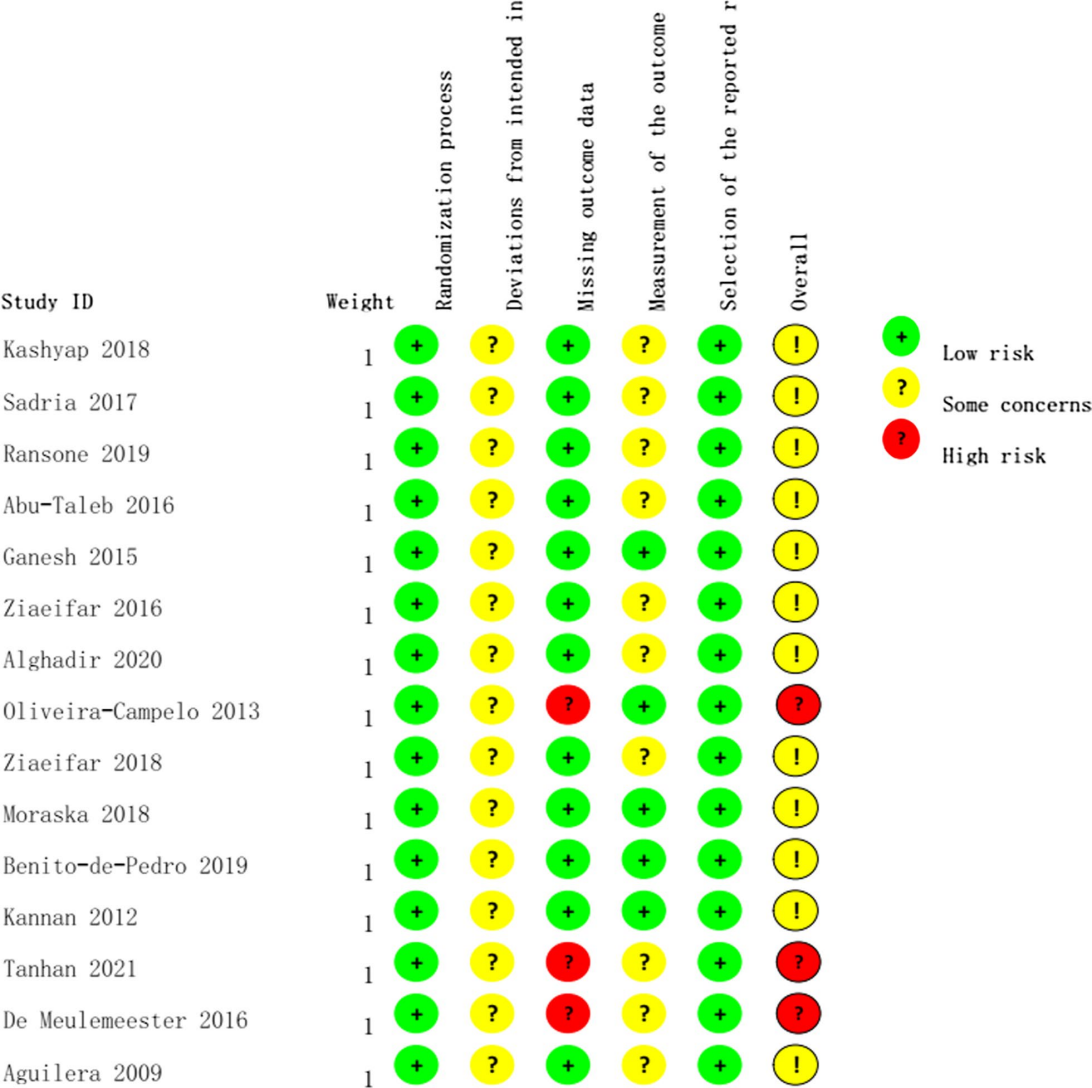
Risk of bias assessment using the ROB 2.0 tool of meta-analysis included RCTs

The description of all included RCTs is provided in Table 1. Among them, 2 RCTs were only included in the systematic review due to a lack of endpoint mean and SD [29, 30]. They were performed in UK [29, 30]. Fifteen RCTs were included in the meta-analysis to obtain a combined result. They were performed worldwide in Turkey [31], Saudi Arabia [32], New Zealand [33], India [13, 34], the USA [35, 36], Iran [37–39], Egypt [40], Spain [41, 42], Belgium [43] and Portugal [44]. All RCTs included meta-analysis reported a consistent baseline. The methodological quality assessment of the 12 studies included in this meta-analysis is shown in Fig. 2. Most studies lacked blinding of the practitioner and/or patients. Most included RCTs reported no dropouts. One RCT reported a < 10% drop out rate [43], two RCTs reported > 15% drop out rates [31, 44], but these three RCTs did not report missing data analysis, such as intention-to-treat analysis.

**Table 1:** The description of included RCTs in systematic review

First author, Year, and location	Population enrolled	Groups and Controls	Characteristic of ischemic compression	Observation time point	Index
Tanhan, 2021, Turkey	75 subjects	Exercise group (n = 25), low level laser group (n = 25), manual pressure release group (n = 25)	Repeated pressure and release on MTrP, until no MTrP tension, three days a week for four weeks	Pre and post treatment	BDI, SF-36, Northwick Park Neck Pain Questionnaire
Kashyap, 2018, India	45 females at 18–30 years	Manual pressure release group (n = 15), muscle energy technique group (n = 15) and control group (n = 15)	Gradually increasing pressure until VAS decrease	Pre and post treatment, 5, 10, 15 days after treatment	PPT, VAS, cervical range of rotation, NDI
De Meulemeester, 2016, Belgium	42 female office workers	Dry needling group (n = 20) and manual pressure group (n = 33)	Pressure on MTrP from 10 N/S to highest tolerable level, 60 s, once a week for 4 weeks	Baseline, after 1 treatment and after 4 treatments, 3 months after treatments	Numeric rating scale, NDI, PPT
Sadria, 2017, Iran	64 subjects at 18–50 years	Pressure and release group (n = 32) and muscle energy group (n = 32)	Pressure or tension with thumb or finger over the MTrP	Pre and post treatment	VAS, Cervical lateral flexion ROM, Upper trapezius thickness
Ransone, 2019, USA	30 subjects	Manual compressive at MTrP group (n = 10), manual compressive at close-proximity within 2.5 cm around MTrP group (n = 10) and control group (n = 10) received sham treatment	Moderate pressure 3 times a week for 4 weeks with each treatment session	Pre and post treatment	PPT
Abu-Taleb, 2016, Egypt	45 subjects	Algometer pressure release (n = 15), pressure release group (n = 15), sham ultrasound group (n = 15)	Repeated pressure and release on MTrPs, until no MTrP tension, three days a week for four weeks	Pre and post treatment	PPT, cervical range of motion
Ganesh, 2015, India	90 subjects with 36 males and 54 females at 19–24 years	Cervical mobilization group (n = 30), ischemic compression group (n = 30) and control group (n = 30)	Ischemic compression to upper trapezius muscle for 5 days	Pre and post treatment, 24 h, 5^th^ day and 2 weeks after treatment	PPT, passive cervical lateral flexion
Aguilera, 2009, Spain	66 subjects with 29 males and 37 females	Ischemic compression group (n = 22), ultrasound group (n = 22), control group (n = 22)	Ischemic compression for 60–90 s	Pre and post treatment	Active ROM, Pressure tolerance, Basal Electrical activity
Ziaeifar, 2016, Iran	32 subjects	Standard group using ischemic compression (n = 17) and experiment group using dry needling (n = 14)	Increasing pressure to MTrP for 3 repetitive times, 1 week	Pre and 3 sessions individually and 2 days after treatmennt	NPS, PPT
Alghadir, 2020, Saudi Arabia	60 subjects at 19–38 years	Group A (n = 20) received all exercise containing hot pack, stretching, ischemic compression, and muscle energy, Group B (n = 20) received exercises except ischemic compression, Group C (n = 20) received exercises except muscle energy therapy	Gradually pressure to MTrP for 90 s	Pre and post treatment	VAS, PPT
Oliveira-Campelo, 2013, Portugal	117 subjects from 18 years	Muscle energy group (n = 23), passive stretching group (n = 23), ischemic compression group (n = 24), placebo group (n = 22), wait and see group (n = 25)	Gradually pressure to MTrP for 90 s	Pre and post treatment	Cervical ROM, PPT, Pressure pain perception
Ziaeifar, 2018, Iran	33 females	Ischemic compression group (n = 17) and dry needling group (n = 16)	Ischemic compression for 90 s	Pre and one week after treatment, 2 weeks and 3 months after treatment	VAS, Disability of arm, hand and shoulder, Northwick park neck pain questionnaire
Moraska, 2018, USA	25 subjects at 18–49 years	Ischemic compression massage on MTrP group (n = 12) and sham ultrasound group (n = 13)	Ischemic compression massage for 6 min	Pre and post treatment, upon probe removal	PPT, blood flow
Benito-de-Pedro, 2019, Spain	34 subjects at 18–75 years	Dry needling group (n = 17) and ischemic compression group (n = 17)	Ischemic compression to MTrP for 90 s	Pre and post treatment	PPT, Thermography
Blikstad, 2008, UK	45 subjects at 18–55 years	Activator trigger point group (n = 15), myofascial band therapy (n = 15) and control group received sham ultrasound (n = 15)	A firm thumb pressure in a slow stroking motion in 1 min	Pre and post treatment	NRS, PPT, cervical ROM, degree of lateral flexion
Kannan, 2012, New Zealand	45 subjects of 22 females and 23 males	Therapeutic ultrasound group (n = 15), laser group (n = 15) and ischemic compression group (n = 15)	Ischemic compression continued 1 and 1 half minute using thumb or strong finger	Pre and post treatment	VAS, ROM, Tenderness
Gemmell, 2008, UK	52 subjects	Ischemic compression group (n = 25) and activator trigger point therapy group (n = 27)	Ischemic compression	Pre and post treatment	NPS, PPT

*VAS* Visual Analogue Scale, *BDI* Beck Depression Inventory, *NRS* numeric rating scale of pain, *NDI* neck disability index, *PPT* pain pressure threshold, *ROM* range of motion, *PT* pain threshold, *MTrP* myofascial trigger points

### Effect of ischemic compression on the PPT index

**Fig. 3:**
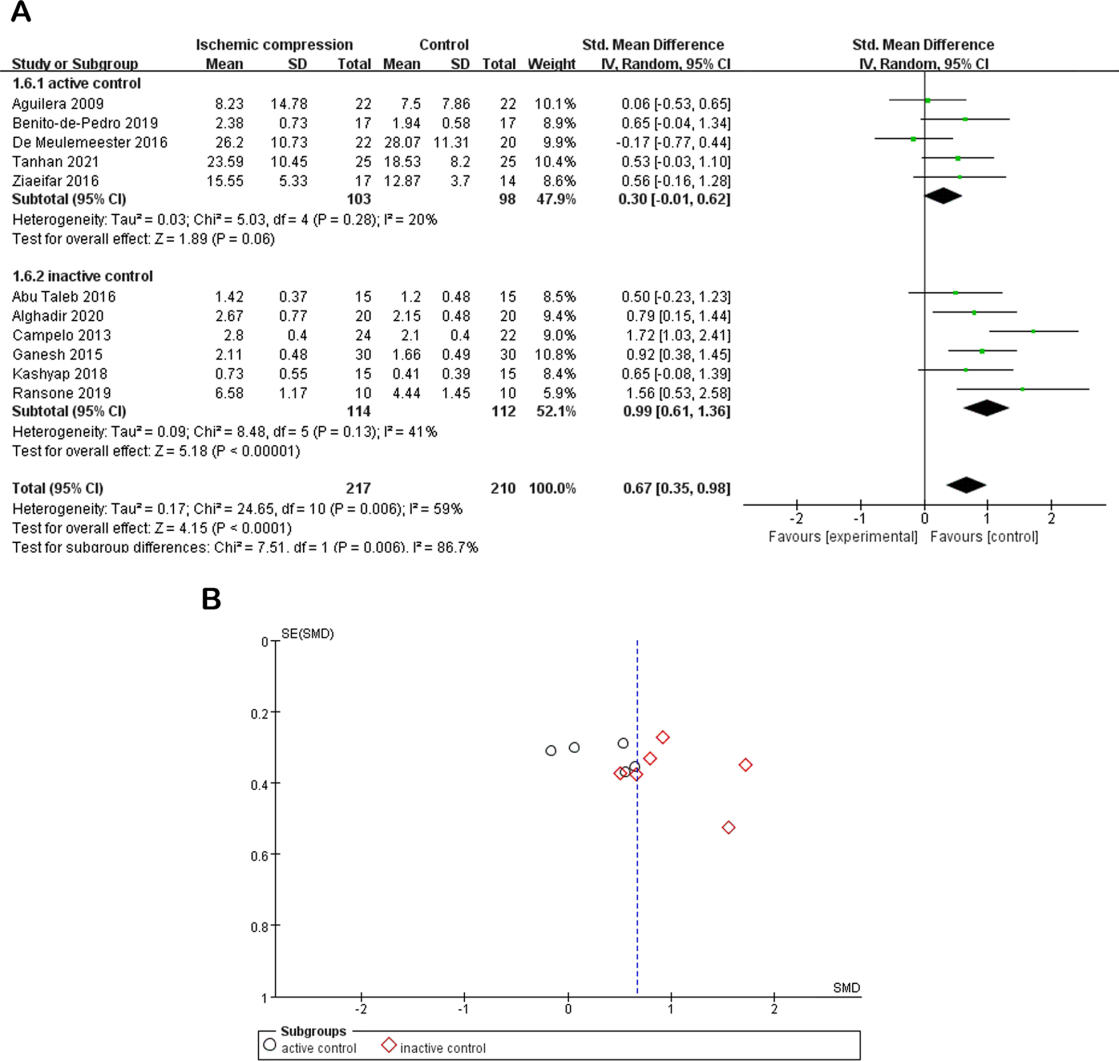
**a** Effect of ischemic compression to PPT values after ischemic treatment within 1 week compared with control on MPS patients. **b** Publication bias

The pain evaluation in MPS was employed using PPT. The trigger point regions, whether active or latent, present a lower PPT than normal muscle [41, 45]. Therefore, PPT is commonly employed to reflect the degree of muscle tolerance to pain of the subject [46]. As shown in Fig. 3, 11 studies and 427 subjects demonstrated statistically significant differences compared with the control at posttreatment (SMD = 0.67, 95% CI [0.35, 0.98], *P* < 0.0001, I^2^ = 59%). A subgroup analysis was performed to explore the comparison effects between ischemic compression and the active control or inactive control group separately, as described previously [47, 48]. As shown in Fig. 3a, there was no statistically significant difference compared with the active control subgroup (SMD = 0.30, 95% CI [− 0.01, 0.62], *P* = 0.06, I^2^ = 20%). Additionally, there was a statistically significant difference compared with the inactive control subgroup (SMD = 0.99, 95% CI [0.61, 1.36], *P* < 0.00001, I^2^ = 41%). These results indicate that ischemic compression enhanced the tolerance to pain in MPS subjects in the inactive control group.

### Effect of ischemic compression on VAS and NRS scores

**Fig. 4:**
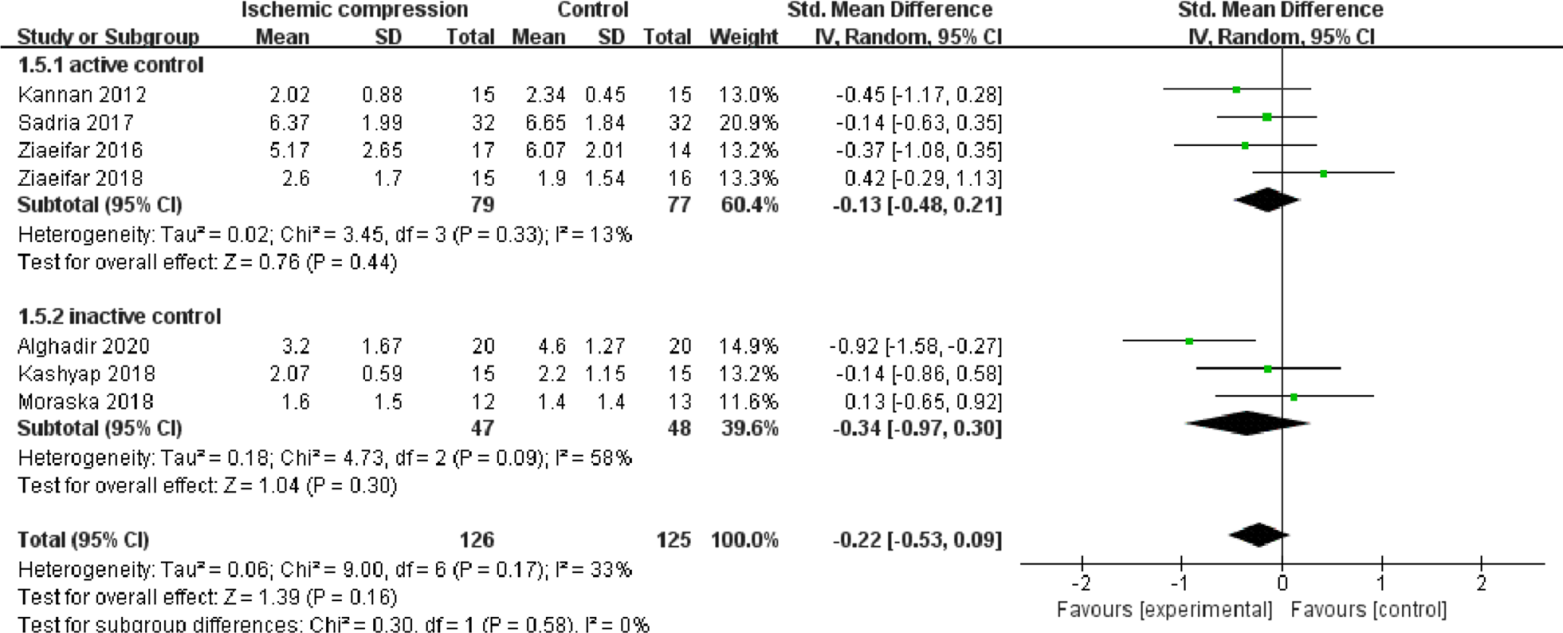
Effect of ischemic compression to VAS or NPS values after ischemic treatment compared with control on MPS patients

The VAS or NRS is generally used for pain assessment to indicate the degree of self-perceived pain of the subject. In this study, as shown in Fig. 4, an analysis of 7 studies and 251 subjects revealed that there was no significant difference in ischemic compression between MPS subjects and controls posttreatment (SMD = − 0.22, 95% CI [− 0.53, 0.09], *P* = 0.16, I^2^ = 33%). There was no statistically significant difference compared with the active control subgroup (SMD = − 0.13, 95% CI [− 0.48, 0.21], *P* = 0.44, I^2^ = 13%). Additionally, there was no statistically significant difference compared with the inactive control subgroup (SMD = − 0.34, 95% CI [− 0.97, 0.30], *P* = 0.30, I^2^ = 58%). These results indicate that ischemic compression did not relieve self-reported pain in MPS subjects compared with both the active or the inactive control group.

### Adverse events

Adverse events were not reported in the included RCTs.

### Publication bias

As shown in Fig. 3b, there was publication bias in the result of PPT index.

## Discussion

A previous review demonstrated that compared with placebo, range of motion may be decreased in MPS patients by some types of manual therapy containing ischemic compression [22]. This study investigated the effectiveness of ischemic compression on pain in MPS patients.

### Study Strengths and comparison with previous meta-analyses

There are some strengths in this systematic review and meta-analysis. The first strength is relative low heterogeneity (< 50%) in the outcomes VAS and two subgroup analysis of PPT, suggesting that the conclusion is solid. Second, compared with 2 previous systematic reviews, one review focused on neck pain, only included neck pain related to myofascial pain and made a qualitative conclusion [20]. However, MPSs are commonly related to headache, neck and shoulder pain, pelvic pain syndromes, and even neuropathic pain [49]. Our meta-analysis added more comprehensive MPS subjects, especially in other pains that clearly stated myofascial pain or the existence of a trigger point in the inclusion criteria. We further performed a meta-analysis. Another review and meta-analysis focused on the range of motion, which is an index of muscle activity [22]. Our meta-analysis focused on self-reported pain and tolerance to pain indices. This helps scientists and clinicians to improve the understanding of ischemic compression to pain reduction in MPS.

### Limitations

There are some limitations in this systematic review and meta-analysis. The first concern is that no studies reported measures of daily activity. Most RCTs only reported the values before and after treatment. The second limitation of this review was the relatively inadequate reporting of subjects included in RCTs. Large-scale RCTs containing over 100 subjects are still lacking. It is suggested that more treating clinicians can be employed or a longer trial period in future research so that more subjects can be included. Thirdly, MPSs were treated using ischemic compression for a short time of less than 1 week in most included studies, as shown in Table 1. Few studies were treated for approximately 1 month, and fewer studies reported 3 months of follow-up. Future studies employing large-scale RCTs with long durations and long-term follow-ups are critical to furthering our knowledge. In addition, 2 RCTs were included in systematic review but excluded in meta-analysis due to lack of data. In their results, part of one suggested few different conclusion [29], one supported conclusions of our meta-analysis [30]. Therefore, these excluded articles do not have a great impact on the analysis results of present meta-analysis.

### Possible intrinsic mechanism

The difference between PPT and VAS attracted the most interest in the present analysis. According to the pathologic hypothesis of MPS [50], the trigger point is caused by the excessive release of acetylcholine from the muscle endplate in this area under various stimuli and injuries, resulting in the shortening of local sarcomere fibers. After sarcomere fibers are shortened, when human muscles move, more blood flow and oxygen supply are needed to maintain normal function, which further aggravates ischemia and hypoxia in the above areas. Pain substances, such as inflammatory factors containing substance P, interleukin-6, bradykinin and interleukin-8, accumulate in the trigger point area [51] and then induce pain termed the “local pain” of the trigger point [52]. As a type of massage, ischemic compression increases the metabolism of the trigger point area through compression and release using mechanical force to alleviate ischemia and hypoxia, reduce the accumulation of inflammatory factors and regulate oxidative stress in the muscle area [53]. This may be the reason why ischemic compression can improve the PPT in muscle. However, VAS is the patient's assessment of pain and the response of the central nervous system to pain. The trigger point, even a latent trigger point, can sensitize nociceptive and non-nociceptive nerve fibers [54] and therefore has a close link to hyperalgesia, allodynia, and referred pain. Pain is transmitted from the local sensory nerve to the dorsal horn neurons and then into the brain [55], eliciting central sensitization, termed “refer pain” [52]. Ischemic compression may not inhibit the sensitization of the central nervous system, which may contribute to the different PPT and VAS results in the present meta-analysis.

An effect on PPT without an effect on pain intensity challenges the diagnosis of myofascial pain syndrome, trigger points maybe not the cause of the painful conditions as myofascial pain syndrome is not a well-defined diagnosis. In addition, it may also can be explained by central sensitization remaining after trigger points were resolved. Central sensitization has become prominent or independent for sustained pain in MPS, therefore pain may persist long although the local trigger point has been dissolved [55]. Referred pain should be considered as a central phenomenon and result of central sensitization [52] or central hyperexcitability [45].

## Conclusion

This meta-analysis explored the pain relief effect of ischemic compression for MPS. Ischemic compression, as a conservative and noninvasive therapy, only enhanced tolerance to pain in MPS subjects compared with inactive control. Furthermore, there was no evidence of benefit for effect of ischemic compression on self-reported pain. The number of currently included subjects was relatively small, so the conclusion may be changed by future studies. Big scale RCTs with more subjects will critical in future.

## Data Availability

This is a review, the dataset supporting the conclusions of this article are extracted from reported literatures and showed within this article.
